# Elucidating Antiangiogenic Potential of *Rauwolfia serpentina*: VEGFR-2 Targeting-Based Molecular Docking Study

**DOI:** 10.1155/2022/6224666

**Published:** 2022-02-14

**Authors:** Adel M. Abuzenadah, Fatin Al-Sayes, Syed Sahajada Mahafujul Alam, Mehboob Hoque, Sajjad Karim, Ibtessam M. R. Hussain, Shams Tabrez

**Affiliations:** ^1^Department of Medical Laboratory Science, Faculty of Applied Medical Sciences, King Abdulaziz University, Jeddah, Saudi Arabia; ^2^King Fahd Medical Research Center, King Abdulaziz University, Jeddah, Saudi Arabia; ^3^Center of Excellence in Genomic Medicine Research (CEGMR), King Abdulaziz University, Jeddah, Saudi Arabia; ^4^Applied Bio-Chemistry Lab, Department of Biological Sciences, Aliah University, Kolkata, India

## Abstract

Angiogenesis plays a critical role in tumorigenesis as it provides the necessary blood supply to the newly grown solid tumor. It helps maintain the tumor microenvironment, promotes tumor development, progression, and metastasis. The vascular epithelial growth factor (VEGF), interacting with the tyrosine kinase receptor VEGFR-2 on endothelial cells, exerts its proangiogenic activity. Hence, targeting the VEGFR-2 signaling is considered a promising strategy to inhibit angiogenesis and thus cancer treatment. This study aims to identify the bioactive compounds derived from the medicinal herb *Rauwolfia serpentina* that effectively binds with VEGFR-2. The bioactive compounds of *R. serpentina* were first screened for their physicochemical properties using the DataWarrior program (version 5.5.0). Finally, 17 compounds that obeyed Lipinski's rule of five and showed good drug-likeness were selected for molecular docking studies. Molecular docking results showed that the ligands ajmalicidine, 1, 2-dihydrovomilenine, rauwolscine, yohimbine, ajmaline, and papaverine interact strongly with the target VEGFR-2 receptor. Hydrogen bonds and hydrophobic interactions stabilized the interactions of these compounds with VEGFR-2. These compounds showed favourable drug-like properties and possess no significant toxicity. Therefore, the findings of this study indicate that the compounds derived from *R. serpentina* can be considered for the development of antiangiogenic drug candidates by targeting VEGFR-2.

## 1. Introduction

Angiogenesis is a multistep process involving a sequence of biological events leading to neovascularization. In various cancers, angiogenesis plays a key role in maintaining the tumor microenvironment and promoting tumor development, progression, and metastasis [1]. The process of angiogenesis is regulated by various pro-and antiangiogenic factors. The vascular epithelial growth factor (VEGF) is an important proangiogenic factor that regulates endothelial cell sprouting and proliferation during vasculogenesis [2]. The VEGF secreted by tumor cells promotes neovascularization that further promotes cancer development. Several members of the VEGF family, including VEGFA, VEGFB, VEGFC, and VEGFD, and the placental growth factor (PLGF) are known to have a role in the regulation of angiogenesis [3]. Among these, the VEGFA isoform is the most functional proangiogenic factor that interacts with the tyrosine kinase receptor VEGFR-2 expressed on endothelial cells [4].

Ligand binding activates VEGFR-2, promoting endothelial cell proliferation and motility, leading to neighboring vessel formation [5]. It is evident that the neovascular tumor endothelial cells display overexpression of VEGFR-2 [6]. The VEGFR-2 overexpression has been observed in cancer cells of various origins including breast, colorectal, urothelial, malignant melanoma, B-cell lymphoma, lung, and others [5]. The VEGFR-2 signaling pathway regulates vascular endothelial cells' permeability, survival, and migration [6]. Therefore, inhibiting the VEGFR-2 signaling in both tumor endothelium and malignant cells is considered a promising target for developing new cancer therapeutics [3].

Conventional cancer therapies such as synthetic anticancer medications and radiation therapy are associated with numerous adverse effects such as severe pain, anemia, fatigue, nausea, vomiting, diarrhoea, alopecia, decreased platelet count, loss of white blood cells, oral ulcers, and adverse radiation-related skin reactions [7]. This has opened up new avenues in the search for alternative anticancer drugs derived from plant sources [8]. Plant-derived phytomedicines are often considered safer than conventional treatments, and they are usually regarded to pose little danger of harm [9]. *Rauwolfia serpentina,* found in the Himalayan mountain range of India and Southeast Asia, is a valuable medicinal herb that belongs to the Apocynaceae family [10, 11]. This plant is known for its pharmacological properties, such as antihypertensive, antibacterial, antifungal, anti-inflammatory, and anticancer activities [12]. The phytochemical constituents of medicinal herbs are the key to their therapeutic potential as they can be utilized as precursors for novel drug development. Moreover, they render superior effects to the plant source as a whole. Therefore, in this study, efforts have been made to identify the bioactive compounds derived from *R. serpentina* that could potentially inhibit angiogenesis by effectively targeting VEGFR-2 by molecular docking.

## 2. Materials and Methods

### 2.1. Physicochemical Properties of Ligands and Their Selection for Molecular Docking

A list of total 25 bioactive compounds of *R. serpentina* was collected from the curated database IMPPAT (Indian Medicinal Plants, Phytochemistry and Therapeutics) (https://cb.imsc.res.in/imppat/home) [13]. These compounds were then assessed by using the DataWarrior program (version 5.5.0) for their various physicochemical properties [14]. Finally, only those compounds that obeyed Lipinski's rule of five were selected for further studies [15].

### 2.2. Preparation of Ligand

The 3D structures of the selected bioactive compounds of *R. serpentina* were downloaded in SDF format from the IMPPAT database, and the atomic coordinates were converted to pdb format using Open Babel GUI [16].

### 2.3. Preparation of Target Protein

The 3D structure of the target protein, human VEGFR-2 (2OH4), was retrieved from Protein Data Bank (https://www.rcsb.org/). This protein was optimized with an MMFF94 force field and prepared for molecular docking by removing heteroatoms (water and ions), adding polar hydrogen, and assigning Kollman charges [17]. To set the active site of the target protein, an appropriate grid box was placed around the cocrystal ligand.

### 2.4. Molecular Docking

The bioactive compounds of *R. serpentina*, selected based on their physicochemical properties and drug-likeness, were docked against the human VEGFR-2 molecular target using the AutoDock 4.2 software [18]. Molecular docking was performed, employing Lamarckian genetic algorithm, with the following set parameters: a starting population of 150 randomly placed individuals, a maximum number of 2,500,000 energy evaluations, a mutation rate of 0.02, and a crossover rate of 0.8. Fifty independent runs were performed for each ligand-target docking. The center of the grid box was set to *X*: 5.396, *Y*: 32.493, and *Z*: 15.884, and the dimensions were *X*: 70, *Y*: 70, and *Z*: 70 with 0.375 Å grid-point spacing. The most suitable binding conformations were selected based on the lowest values of free energy of binding (Δ*G*) and inhibition constant (Ki). The molecular interactions of the selected ligands with the target protein at their lowest binding energy poses were analysed using LigPlot+ v.2.2.4 [19].

## 3. Results and Discussion

*R. serpentina* is traditionally used for its various health benefits. Recent studies also report the anticancer properties of the plant [12]. In this study, we investigated the phytoconstituents of *R. serpentina* against human VEGFR-2 to identify its potential inhibitors by using a molecular docking approach. The molecular docking technique can be reliably used to predict the potential drug candidates against certain target proteins in pursuit of therapeutic development of various diseases [20–22]. There are a total of 25 bioactive compounds of *R. serpentina* retrieved from the IMPPAT database, and their physicochemical properties were studied using the DataWarrior program. Only those compounds that obeyed Lipinski's rule of five were selected for molecular docking studies against the VEGFR-2 target [15]. It was found that 17 out of total 25 compounds obeyed Lipinski's rule of five and showed good drug-likeness scores. The detailed physicochemical properties of these selected compounds are shown in Table 1. These compounds also possessed none of the mutagenic, tumorigenic, irritant, or any adverse effects on reproductive health. Moreover, the physicochemical properties like polar surface area and rotatable bonds were also within permissible limits of 140 Å^2^ and 10, respectively. Other eight compounds that showed violation in at least any of the rule criteria have been eliminated from further studies.

The selected compounds were then investigated to identify the most potent antiangiogenic drug candidates by targeting VEGFR-2 receptor using a molecular docking approach. The redocking experiment was performed before molecular docking studies with the selected ligands to validate the docking technique and algorithm. The root-mean-square deviation value was found to be less than 2 Å between the native cocrystal and the docked positions. This suggests that the docking procedures and parameters used in this work can accurately predict the compounds' natural conformations [23]. In this study, the threshold binding energy (Δ*G*) was set at −9.0 kcal/mol, and the best ligands were chosen based on the binding energy of −9.0 kcal/mol or greater negative values. Out of the 17 molecules selected after the physicochemical screening, six major compounds such as ajmalicidine, 1, 2-dihydrovomilenine, rauwolscine, yohimbine, ajmaline, and papaverine showed binding energy greater than the threshold value, i.e., ≤ −9.0 kcal/mol. Detailed molecular interactions of these ligands with VEGFR-2 were studied further as depicted in Table 2.

**Figure 1:**
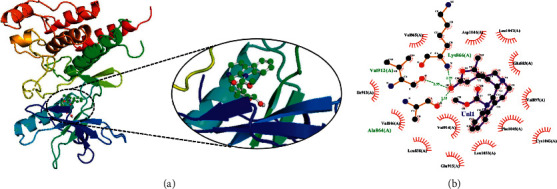
The binding pattern of ajmalicidine derived from *R. serpentina* with VEGFR-2. Panel (a) shows the 3D interaction of VEGFR-2 with ajmalicidine visualized using PyMol. Panel (b) represents the 2D image of the molecular interactions between the protein and ligand generated by Ligplot+ v.2.2.4. The green dashed lines and the spiked red arcs represent the hydrogen bonds with bond distance and the residues involved in hydrophobic interactions, respectively.

The ligand ajmalicidine was best docked to VEGFR-2 with ΔG of −10.08 kcal/mol and an inhibition constant (Ki) of 41.06 nM. It showed strong interaction with VEGFR-2 through three hydrogen bonds through Ala864, Lys866, and Val912 and multiple hydrophobic interactions through Leu838, Val846, Val865, Glu883, Val897, Ile913, Val914, Glu915, Leu1033, Cys1043, Asp1044, and Phe1045. These molecular interactions of ajmalicidine with VEGFR-2 are shown in Figure 1. Therapeutic application of this indole alkaloid derived from *R. serpentina* is not yet reported in cancer treatment. Our finding suggests a promising potential of this compound and warrants further exploration for its therapeutic use.

**Figure 2:**
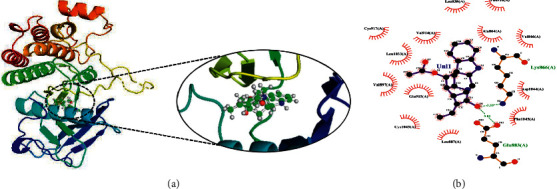
The binding pattern of 1, 2-dihydrovomilenine derived from *R. serpentina* with VEGFR-2. Panel (a) shows the 3D interaction of VEGFR-2 with 1, 2-dihydrovomilenine visualized using PyMol. Panel (b) represents the 2D image of the molecular interactions between the protein and ligand generated by Ligplot+ v.2.2.4. The green dashed lines and the spiked red arcs represent the hydrogen bonds with bond distance and the residues involved in hydrophobic interactions, respectively.

Another compound, 1, 2-dihydrovomilenine was best docked to VEGFR-2 with a Δ*G* of −10.06 kcal/mol and Ki of 17.06 nM. It showed strong interaction with VEGFR-2 through two hydrogen bonds with Lys866, Glu883, and multiple hydrophobic interactions via Leu838, Val846, Ala864, Leu887, Val897, Val914, Glu915, Phe916, Cys917, Leu1033, Cys1043, Asp1044, and Phe1045. The molecular interactions of 1,2-dihydrovomilenine with the VEGFR-2 are shown in Figure 2.

**Figure 3:**
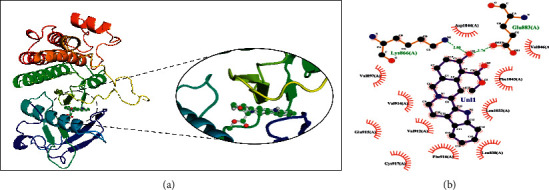
The binding pattern of rauwolscine derived from *R. serpentina* with VEGFR-2. Panel (a) shows the 3D interaction of VEGFR-2 with rauwolscine visualized using PyMol. Panel (b) represents the 2D image of the molecular interactions between the protein and ligand generated by Ligplot+ v.2.2.4. The green dashed lines and the spiked red arcs represent the hydrogen bonds with bond distance and the residues involved in hydrophobic interactions, respectively.

**Figure 4:**
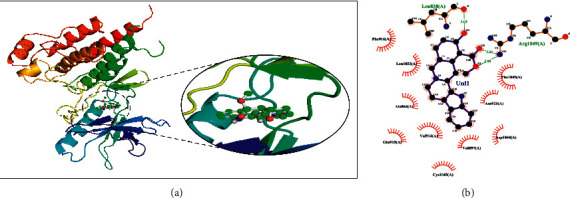
The binding pattern of yohimbine derived from *R. serpentina* with VEGFR-2. Panel (a) shows the 3D interaction of VEGFR-2 with yohimbine visualized using PyMol. Panel (b) represents the 2D image of the molecular interactions between the protein and ligand generated by Ligplot+ v.2.2.4. The green dashed lines and the spiked red arcs represent the hydrogen bonds with bond distance and the residues involved in hydrophobic interactions, respectively.

Rauwolscine was best docked to VEGFR-2 with a ΔG of −10.4 and Ki of 23.79 nM. The interaction of rauwolscine with VEGFR-2 as shown in Figure 3 depicts the formation of two hydrogen bonds with Lys866 and Glu883 and hydrophobic interactions with residues Leu838, Ala864, Val897, Val912, Val914, Glu915, Phe916, Cys917, Leu1033, Asp1044, and Phe1045. The ligand yohimbine was best docked to VEGFR-2 with Δ*G* of −9.7 kcal/mol and Ki of 78.00 nM and established two hydrogen bonds through Leu838 and Arg1049. Furthermore, hydrophobic interactions were formed involving the residues Ala864, Val897, Val914, Glu915, Phe916, Asn921, Leu1033, Cys1043, Asp1044, and Phe1045 (Figure 4). Both these compounds, rauwolscine and yohimbine are known antagonists of *α*2-adrenoceptor and are reported to inhibit the proliferation of breast cancer cells [24, 25]. Moreover, the yohimbine was also shown to exhibit an inhibitory effect against human pancreatic cancer cell proliferation by inducing apoptosis [26].

**Figure 5:**
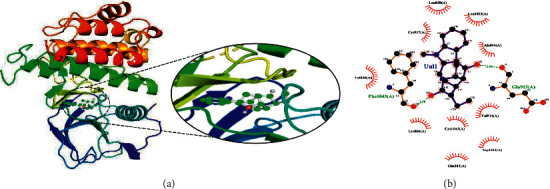
The binding pattern of ajmaline derived from *R. serpentina* with VEGFR-2. Panel (a) shows the 3D interaction of VEGFR-2 with ajmaline visualized using PyMol. Panel (b) represents the 2D image of the molecular interactions between the protein and ligand generated by Ligplot+ v.2.2.4. The green dashed lines and the spiked red arcs represent the hydrogen bonds with bond distance and the residues involved in hydrophobic interactions, respectively.

**Figure 6:**
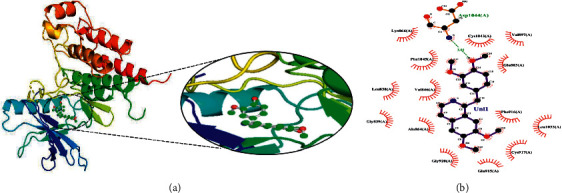
The binding pattern of papaverine derived from *R. serpentina* with VEGFR-2. Panel (a) shows the 3D interaction of VEGFR-2 with papaverine visualized using PyMol. Panel (b) represents the 2D image of the molecular interactions between the protein and ligand generated by Ligplot+ v.2.2.4. The green dashed lines and the spiked red arcs represent the hydrogen bonds with bond distance and the residues involved in hydrophobic interactions, respectively.

Another ligand that showed strong binding to VEGFR-2 with binding energy within the set threshold is ajmaline. It was best docked with Δ*G* of −9.44 kcal/mol and Ki of 121.28 nM and established only one hydrogen bond through Glu915. Moreover, this interaction was further stabilized by hydrophobic interactions with the active residues Leu838, Val846, Ala864, Lys866, Glu883, Val914, Cys917, Leu1033, Cys1043, and Asp1044. The molecular interactions of ajmaline with the VEGFR-2 are shown in Figure 5. Apart from all these *R. serpentina* ligands, papaverine also showed binding with VEGFR-2 with ΔG of −9.06 kcal/mol and Ki of 230.32 nM and established one hydrogen bond through Asp1044. Moreover, hydrophobic interactions were also formed with residues Leu838, Gly839, Val846, Ala864, Lys866, Glu883, Val897, Glu915, Phe916, Cys917, Gly920, Leu1033, Cys1043, and Phe1045. The molecular interactions of papaverine with the VEGFR-2 are shown in Figure 6. The papaverine is shown to exhibit anticancer activity against various types of cancer [27, 28].

## 4. Conclusion

**Figure 7:**
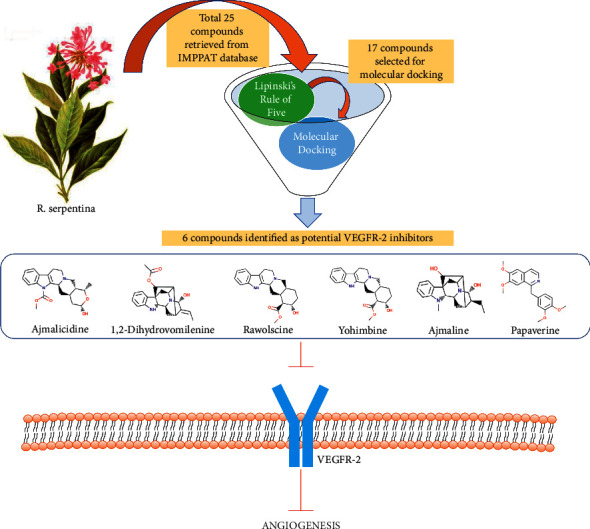
Schematic representation of identification of potential VEGFR-2 inhibitors derived from *R. serpentina* by molecular docking.

The present study revealed the possible constituents of *R. serpentina* that can potentially bind with and inhibit VEGFR-2. This study further provides structural insights into the possible modes of interaction of the ligands with the target. There are six compounds *viz.* ajmalicidine, 1, 2-dihydrovomilenine, rauwolscine, yohimbine, ajmaline, and papaverine identified to show strong interactions with VEGFR-2 with high binding energy and low Ki values. The overall scheme of identification of potential VEGFR-2 inhibitors derived from *R. serpentina* is shown in Figure 7. All these compounds were found to form a good number of hydrogen bonds and hydrophobic interactions with VEGFR-2 indicating a stable interaction. All the compounds derived from *R. serpentina* were initially screened for drug-likeliness and physicochemical properties and considered only those with drug-like properties for molecular docking studies. Therefore, the identified six compounds can be considered promising leads for designing a specific VEGFR-2 inhibitor. Targeting VEGFR-2 by the identified *R. serpentina* compounds will have implications in inhibiting angiogenesis in various forms of cancer. However, further studies on *in vitro* as well as *in vivo* models would be required to validate their therapeutic application in cancer treatment.

## Figures and Tables

**Table 1:** Physicochemical properties of the selected bioactive compounds derived from *R. serpentina*.

Sl. no.	Ligand name	Structure	Total mol. weight	cLogP	cLogS	H-acceptors	H-donors	Polar surface area	Drug-likeness	Mutagenic	Tumorigenic	Reproductive effective	Irritant	Rotatable bonds
1	Isoajmaline	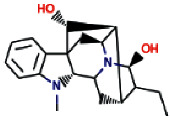	326.438	1.791	−3.484	4	2	46.94	3.4513	None	None	None	None	1
2	Ajmalicidine	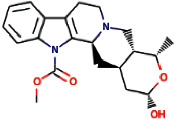	370.447	3.0403	−3.438	6	1	63.93	2.2403	None	None	None	None	2
3	Sarpagine	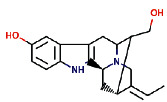	310.396	2.4395	−2.632	4	3	59.49	2.0345	None	None	None	None	1
4	Ajmalinimine	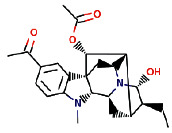	410.512	2.1468	−4.578	6	1	70.08	3.6292	None	None	None	None	4
5	Indoline	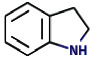	119.166	1.3351	−2.025	1	1	12.03	0.19917	None	None	None	None	0
6	Indobinine	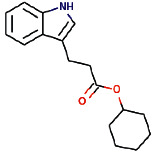	271.359	3.6202	−4.091	3	1	42.09	−7.331	None	None	None	None	5
7	1,2-Dihydrovomilenine	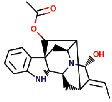	352.433	1.8244	−3.654	5	2	61.8	1.1872	None	None	None	None	2
8	Alloyohimbine	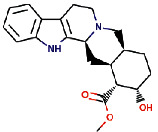	354.448	2.3512	−3.065	5	2	65.56	1.5035	None	None	None	None	2
9	Ajmaline	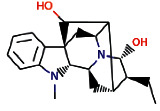	326.438	1.791	−3.484	4	2	46.94	3.4513	None	None	None	None	1
10	Ajmalicine	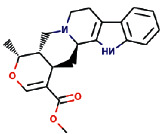	352.433	2.2674	−3.141	5	1	54.56	2.6043	None	None	None	None	2
11	Papaverine	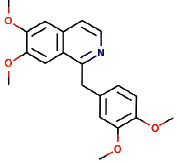	339.39	3.443	−4.234	5	0	49.81	−1.7454	None	None	None	None	6
12	Ajmaline	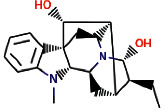	326.438	1.791	−3.484	4	2	46.94	3.4513	None	None	None	None	1
13	Rauwolscine	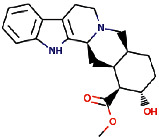	354.448	2.3512	−3.065	5	2	65.56	1.5035	None	None	None	None	2
14	Isorauhimbine	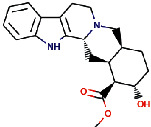	354.448	2.3512	−3.065	5	2	65.56	1.5035	None	None	None	None	2
15	3,4,5-Trimethoxybenzoic acid	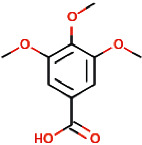	212.2	0.9347	−1.683	5	1	64.99	−1.597	None	Low	None	None	4
16	Benzyl 3-indole propionate	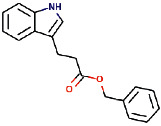	279.338	3.4826	−3.84	3	1	42.09	−3.2296	None	None	None	None	6
17	Yohimbine	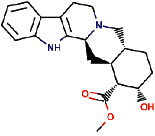	354.448	2.3512	−3.065	5	2	65.56	1.5035	None	None	None	None	2

**Table 2:** The interaction parameters of selected bioactive compounds of *R. serpentina* docked against VEGFR-2.

Sl. no.	Ligand name	Binding energy, Δ*G* (Kcal/mol)	Inhibition constant (Ki) (nM)	Interactive residues
1	Ajmalicidine	−10.08	41.06	Ala864, Lys866, Val912, Leu838, Val846, Val865, Glu883, Val897, Ile913, Val914, Glu915, Leu1033, Cys1043, Asp1044, and Phe1045
2	1, 2-Dihydrovomilenine	−10.6	17.06	Lys866, Glu883, Leu838, Val846, Ala864, Leu887, Val897, Val914, Glu915, Phe916, Cys917, Leu1033, Cys1043, Asp1044, and Phe1045
3	Rauwolscine	−10.4	23.79	Lys866, Glu883, Leu838, Ala864, Val897, Val912, Val914, Glu915, Phe916, Cys917, Leu1033, Asp1044 and Phe1045
4	Yohimbine	−9.7	78.00	Leu838, Arg1049, Ala864, Val897, Val914, Glu915, Phe916, Asn921, Leu1033, Cys1043, Asp1044, and Phe1045
5	Ajmaline	−9.44	121.28	Glu915, Leu838, Val846, Ala864, Lys866, Glu883, Val914, Cys917, Leu1033, Cys1043, and Asp1044
6	Papaverine	−9.06	230.32	Asp1044, Leu838, Gly839, Val846, Ala864, Lys866, Glu883, Val897, Glu915, Phe916, Cys917, Gly920, Leu1033, Cys1043, and Phe1045

## Data Availability

The data used to support the findings of this study are available upon request to the corresponding author.
